# Spatiotemporal Trends, Sources and Ecological Risks of Heavy Metals in the Surface Sediments of Weitou Bay, China

**DOI:** 10.3390/ijerph18189562

**Published:** 2021-09-10

**Authors:** Qiuming Chen, Faming Huang, Anran Cai

**Affiliations:** Third Institute of Oceanography, Ministry of Natural Resources, Xiamen 361005, China; chenqiuming@tio.org.cn (Q.C.); 18060207886@163.com (A.C.)

**Keywords:** heavy metal, surface sediment, pollution, spatiotemporal variation, ecological risk, source

## Abstract

Heavy metals are extremely harmful materials to marine ecosystems and human health. To determine the anthropogenic contributions and ecological risks in Weitou Bay, China, the spatiotemporal variations in the concentrations of heavy metals in surface sediment were investigated during spring 2008 and 2017. The results indicated that high concentrations of pollutants were generally located near the river mouths and along the coast of industrial areas. Principal component analysis indicated that heavy metal contents were mainly affected by industrial waste drainage, urban development, natural weathering and erosion, and interactions between organic matter and sulfides. The potential ecological risk assessment demonstrated that, in 2008, 82% of the sampling sites were at low risk, while 18% were at moderate risk. The situation had deteriorated slightly by 2017, with 73%, 18%, and 9% of stations in Waytou Bay at low, moderate, and very high risk, respectively. Cd was the most harmful metal, followed by Hg. These two elements accounted for more than 80% of the potential ecological risk index (*RI*) value. The present work analyzed the source of heavy metals, identified the major pollution elements and high risk areas, and provides guidance for pollution control and ecological restoration in Weitou Bay.

## 1. Introduction

As regions of active land–sea interactions, coastal areas are considered to be sinks for various contaminants derived from natural source and anthropogenic activities [1]. Along with rapid economic growth in coastal regions, an increasing number of pollutants are discharged into the ocean, leading to serious environmental problems and potential threats to aquatic organisms and marine ecosystems [2,3]. 

Unlike some organic pollutants, heavy metals do not degrade easily. Due to their persistence, toxicity, bioaccumulation, and non−biodegradability, heavy metals have been acknowledged as extremely harmful materials to marine ecosystems and human health [4,5]. Owing to the effects of adsorption, flocculation, and subsidence, a considerable amount of heavy metal pollutants discharged into seawater are transferred to the sediment, while only a small part remains dissolved in the water [6]. Nevertheless, under complex physicochemical and dynamic conditions, heavy metals can be released from surface sediments into the seawater [7]. Therefore, sediment becomes not only a reservoir for heavy metals in the ocean, but also an indicator of contamination in the marine environment. To a certain extent, the distribution of heavy metals in sediments reflects the pollution level of the marine environment and is commonly used to determine the source(s) of the pollutants [8]. Studying the distributions and pollution status of heavy metals and their controlling mechanisms under the influence of anthropogenic activity provides a basis for pollution control and environmental protection.

Weitou Bay is located in Quanzhou City in Fujian Province, which is an important location of economic growth in southeastern China. From 2008 to 2017, the GDP in Quanzhou increased from 270.5 billion Chinese RMB (CNY) to 754.8 billion, and the resident population increased by 900,000 people [9,10]. Along with accelerated industrialization and urbanization during recent decades, an increasing number of factories have been constructed in the coastal area of Weitou Bay, including textile factories, bleaching and dyeing factories, tanneries factories, and electroplating factories. Large quantities of pollutants from land-based sources, including continental runoff, industrial effluent, and domestic wastewater, are released into Weitou Bay and ultimately accumulate in seafloor deposits [11]. Since 2008, environmental protection and restoration measures have been conducted in Weitou Bay, including upstream river regulation, marine aquaculture dredging, and sewage interception, which may improve the quality of the marine environment. In contrast, the rapid industrialization, urbanization, reclamation, and enhancement of the port’s throughput capacity have increased pressure on the marine ecosystem. 

Some studies of heavy metals in Weitou Bay have been conducted, but these were mainly limited in their spatial distributions, sources, and potential ecological risks [11,12,13]. Studies of spatiotemporal variations in heavy metals in this area are currently lacking. In order to explore the combined effects of environment protection and increased anthropogenic pressure on the marine environment, we investigated the spatiotemporal variations in heavy metals in the surface sediments of Weitou Bay under complex driving forces over 10 years (in 2008 and 2017). 

The main goals of this study included: (1) determining the temporal variations in heavy metals in the surface sediment; (2) determining the spatial distributions of heavy metals; (3) exploring the main sources of heavy metals and analyzing the factors that cause spatiotemporal changes in pollution; (4) assessing the potential ecological risk levels, identifying the main ecological risk factors, and making recommendations for pollution control and ecological restoration in the bay.

## 2. Materials and Methods

### 2.1. Study Area

**Figure 1:**
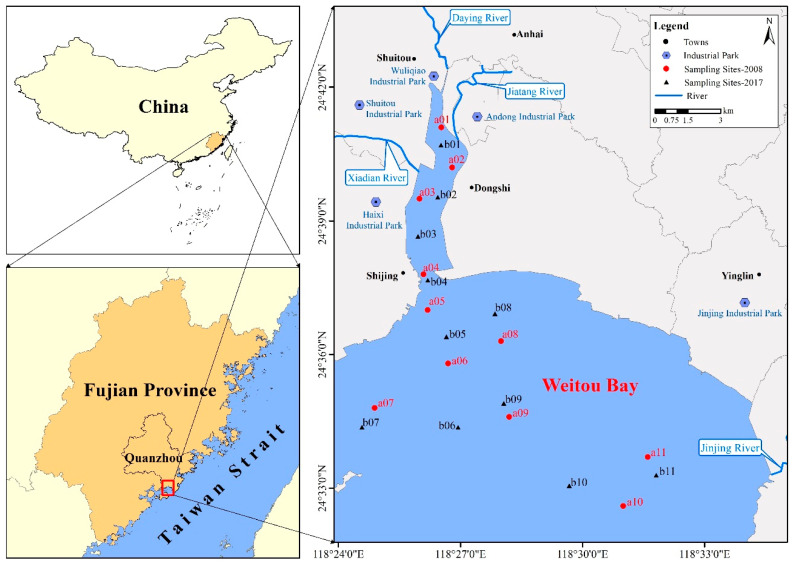
Location of the study area in Weitou Bay, China.

Weitou Bay is a semi−enclosed bay located in southeastern Fujian Province, China, to the west of the Taiwan Strait (Figure 1), and is the beginning of the ancient Maritime Silk Road in China. The bay is characterized by regular semi−diurnal tides with an average tidal range of 4.19 m [14] and does not freeze, owing to the subtropical monsoon climate in the region. Weitou Bay is trumpet-shaped, with a narrow northern bay (inner bay) that extends inland. The inner bay has an 800 m mouth and is approximately 9 km long, 1.5 km wide, and covers 13.1 km^2^, of which 9.8 km^2^ comprises intertidal flats [13]. The inner bay receives continuous inputs of land−based pollutants from the Daying, Xiadian, and Jiatang Rivers, and the seabed in this area has a high sedimentation rate [11]. 

The southern part of Weitou Bay (outer bay) has a relatively wider mouth and deeper water depths. The hydrodynamic conditions due to vertical and horizontal mixing gradually increase from the inner bay to the outer bay [8]. The outer bay is 12 km wide in the N–S direction and 22 km wide in the E–W direction, with an area of 279.9 km^2^, of which 58.1 km^2^ is intertidal flats. Two deep grooves ~15 m in depth are located in the middle of the outer bay [15,16].

Weitou Bay is influenced by human activity (sewage and industrial discharge, harbor activities, reclamation), which have increased its environmental risk, decreased the tidal volume, caused the water to become eutrophic, and resulted in huge amounts of pollutants, including heavy metals being deposited in the sediment. These factors all pose threats to aquatic organisms and the marine ecosystem in the bay [12,13,17].

### 2.2. Sampling and Analyses

Surface sediment (0–5 cm) samples were collected from Weitou Bay during the spring of 2008 and 2017 using a stainless−steel grab sampler (Figure 1). The samples were then placed into sealed polyethylene bags and stored at 4 °C until analysis. Total organic carbon (TOC), sulfide, and heavy metal (Cu, Pb, Zn, Cd, Cr, Hg, and As) concentrations were analyzed following standard Chinese marine monitoring methods (GB 17378.5−2007) [18].

Prior to analysis, the samples were dried at 60 °C, ground to pass through a 160−mesh sieve, and fully mixed. To determine the contents of Cu, Pb, Zn, Cd, and Cr, 0.1 g of the dry samples was digested with a solution of HNO_3_+HClO_4_+HCl (6:2:1 by volume) in glass beakers on an electric hotplate for 2 h. The compositions and corresponding concentrations of the digested samples were then analyzed using atomic absorption spectrometry (Jenna Zeenit 6000). For the Hg and As analyses, 0.1 g of each sample was digested with chloroazotic acid (HNO_3_+HCl, 3:1 by volume) for 2 h, then analyzed using atomic fluorescence spectrometry (AFS−920). TOC concentrations were determined using the potassium dichromate oxidation method [18]. Sulfide concentrations were analyzed using the iodine method [18].

For data quality control, standard reference materials (GBW07314, from the National Research Center for Certified Reference Materials, Beijing, China) were analyzed using the above method, and the recovery rates of the monitoring factors ranged from 95% to 110%. The determinations had a precision (represented by the average relative standard deviation (RSD) of parallel samples) of better than 5.0%. The quality control measurement results indicated that the determination accuracy met the requirements of this study (an average RSD of parallel samples less than 5%, and recovery rates of the monitoring factors between 90~110% [18]).

### 2.3. Potential Ecological Risk Assessment of Sediment

The potential ecological risk index (*RI*) was used to obtain a comprehensive assessment of ecological pressure from heavy metals. The *RI* was calculated using the following Equations [19]:

(1) $$C_{f}^{i}=C^{i}/C_{n}^{i}$$

(2) $$E_{r}^{i}=T_{r}^{i}\times C_{f}^{i}$$

(3) $$RI=\sum_{i=1}^{m}E_{r}^{i}$$

where *i*, *f*, and *m* denote a specific metal *i* in the sediment sample, fraction, and total number of metals analyzed in the sediment sample, respectively. $C_{f}^{i}$ is the contamination factor for an individual metal. $C^{i}$ is the concentration of metal *i* in the sample and $C_{n}^{i}$ is the background value for metal *i*. In this study, the heavy metals concentrations in the coastal soil in Fujian Province served as the background values [20] (Table 1). $E_{r}^{i}$ is the potential ecological risk factor for a single heavy metal. $T_{r}^{i}$ is the toxicity factor for heavy metal *i*, representing its toxicity levels and the sensitivity towards bio-organisms. In this study, Hakanson’s standard toxicity factors were adopted; the values for the toxicity factors of Cu, Pb, Zn, Cd, Cr, Hg, and As are 5, 5, 1, 30, 2, 40, and 10, respectively [19,21]. 

The $E_{r}^{i}$ and *RI* values are classified into five and four degrees of ecological risk, respectively. The classifications are as follows: (1) $E_{r}^{i}$ < 40, low ecological risk; 40 < $E_{r}^{i}$ <80, moderate ecological risk; 80< $E_{r}^{i}$ <160, considerable ecological risk; 160< $E_{r}^{i}$ <320, very high ecological risk; $E_{r}^{i}$ ≥ 320, disastrous ecological risk; (2) RI < 150, low ecological risk; 150 < RI < 300, moderate ecological risk; 300 < RI < 600, considerable ecological risk; RI ≥ 600, very high ecological risk [22].

### 2.4. Statistical Analyses

The SPSS software package (version 20.0) was used to determine the Pearson correlation coefficients of all tested factors, and principal component analysis (PCA) was conducted to explore the potential pollution sources (natural or artificial) and their elemental characteristics. PCA is commonly used to identify potential pollution sources, distinguish natural and anthropogenic contributions, and indicate biogeochemical processes related to heavy metals. In this study, principal components were performed based on the correlation matrix and extracted if their eigenvalues were greater than 1.0. We then used ArcGIS 10.0 (Environmental Systems Research Institute, inc.) with the Kriging spatial interpolation method to quantitatively determine the spatial distributions of the heavy metals.

## 3. Results and Discussion

### 3.1. Spatiotemporal Distributions of Heavy Metals

#### 3.1.1. Temporal Variations and Trends

**Figure 2:**
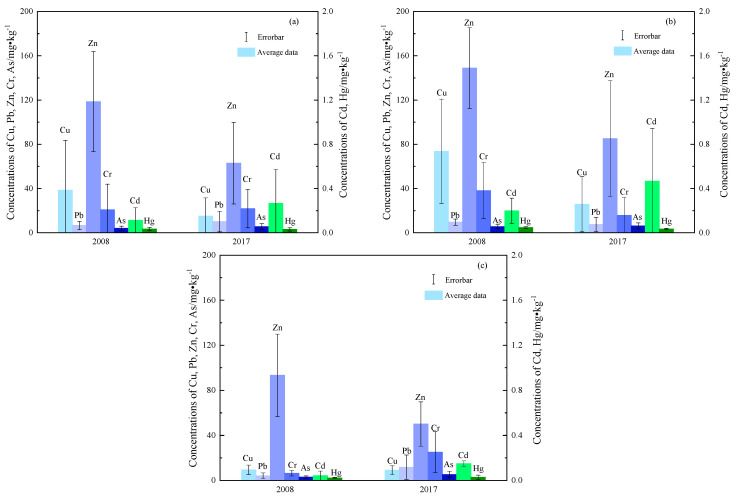
Heavy metal concentrations of the surface sediment from (**a**) the entire Weitou Bay, (**b**) inner Weitou Bay, (**c**) outer Weitou Bay.

The heavy metal concentrations in the Weitou Bay surface sediment are presented in Table 1 and Figure 2. For the seven heavy metals measured in the surface sediments, the mean concentrations of Cu and Zn decreased from 2008 to 2017, while the Cd, Pb, and As concentrations increased and the Hg and Cr concentrations did not vary significantly (Figure 2a). Specifically, the mean concentrations and ranges of Cu and Zn decreased from 38.7 mg·kg^−1^ (3.5–155.0 mg·kg^−1^) and 118.6 mg·kg^−1^ (23.4–213.0 mg·kg^−1^) to 15.2 mg·kg^−1^ (3.1–62.8 mg·kg^−1^) and 62.9 mg·kg^−1^ (20.8–161.0 mg·kg^−1^), respectively. Although the concentrations of Cd, Pb, and As increased from 2008 to 2017, As concentrations increased less than those of Cd and Pb. Cd increased 135% from 0.113 mg·kg^−1^ (0.016–0.384 mg·kg^−1^) to 0.265 mg kg^−1^ (0.123–1.180 mg·kg^−1^). Pb increased 57% from 6.6 mg·kg^−1^ (1.8–11.9 mg·kg^−1^) to 10.2 mg·kg^−1^ (0.1–26.3 mg·kg^−1^). However, As increased 37% from 4.1 mg·kg^−1^ (1.3–8.5 mg·kg^−1^) to 5.6 mg·kg^−1^ (2.1–10.0 mg·kg^−1^). In addition, both Hg and Cr exhibited small changes from 2008 to 2017. The mean Hg concentration was 0.034 mg·kg^−1^ (0.017–0.060 mg·kg^−1^) in 2008 and 0.030 mg·kg^−1^ (0.012–0.054 mg·kg^−1^) in 2017. Correspondingly, the mean Cr concentration was 20.87 mg·kg^−1^ (2.61–78.40 mg·kg^−1^) in 2008 and 21.77 mg·kg^−1^ (5.20–56.30 mg·kg^−1^) in 2017.

It can be judged from the RSD value (Table 1) that the difference in the spatial distribution of Cu was the most significant among the seven heavy metals, followed by Cd and Cr, while the difference in the spatial distribution of Pb, Zn, Hg, and As was relatively small. On the other hand, as shown in Figure 2, the mean concentrations of heavy metals in the outer bay were lower than those in the inner bay in 2008. In 2017, except for Pb and Cr, the averages of other elements were still lower than those in the inner bay. Overall, the outer bay was less polluted by heavy metals than the inner bay.

The 2008 average concentrations of Cu (38.7 mg·kg^−1^), Zn (118.6 mg·kg^−1^), and Cd (0.113 mg·kg^−1^) in the surface sediments of Weitou Bay were higher than the environmental background values. However, since Cu (15.2 mg·kg^−1^) and Zn (62.9 mg·kg^−1^) decreased in 2017, only the mean Cd concentration (0.265 mg·kg^−1^) was higher than the environmental background value. In contrast, in both 2008 and 2017, the mean concentrations of the other heavy metals (Pb, Cr, Hg, and As) were below the environmental background values.

#### 3.1.2. Spatial Distributions of Heavy Metals

**Figure 3:**
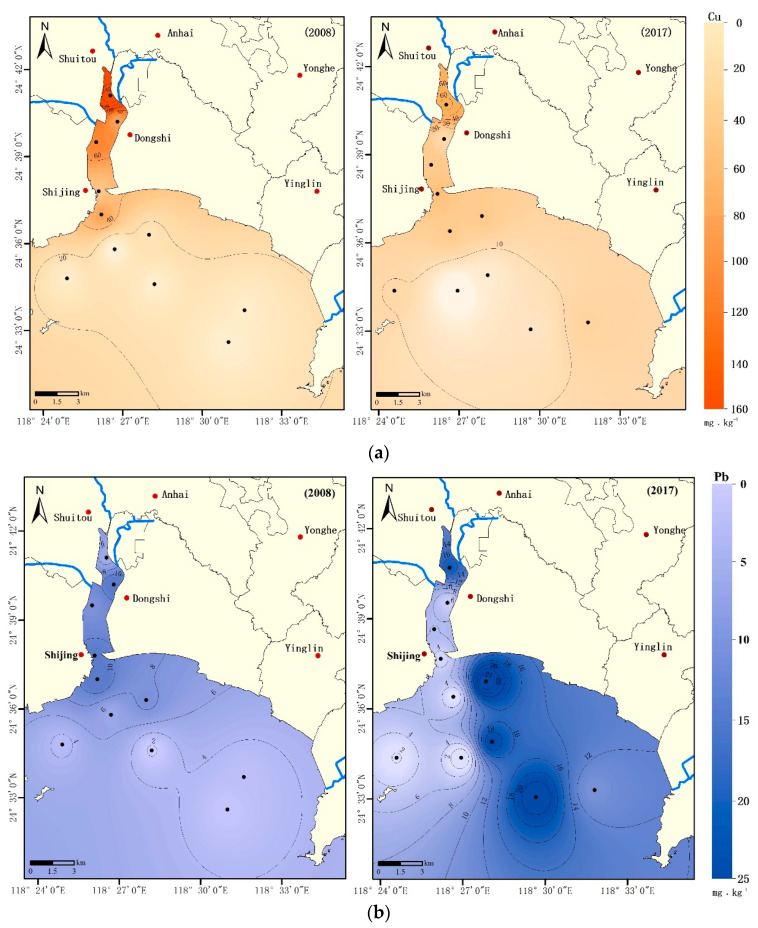

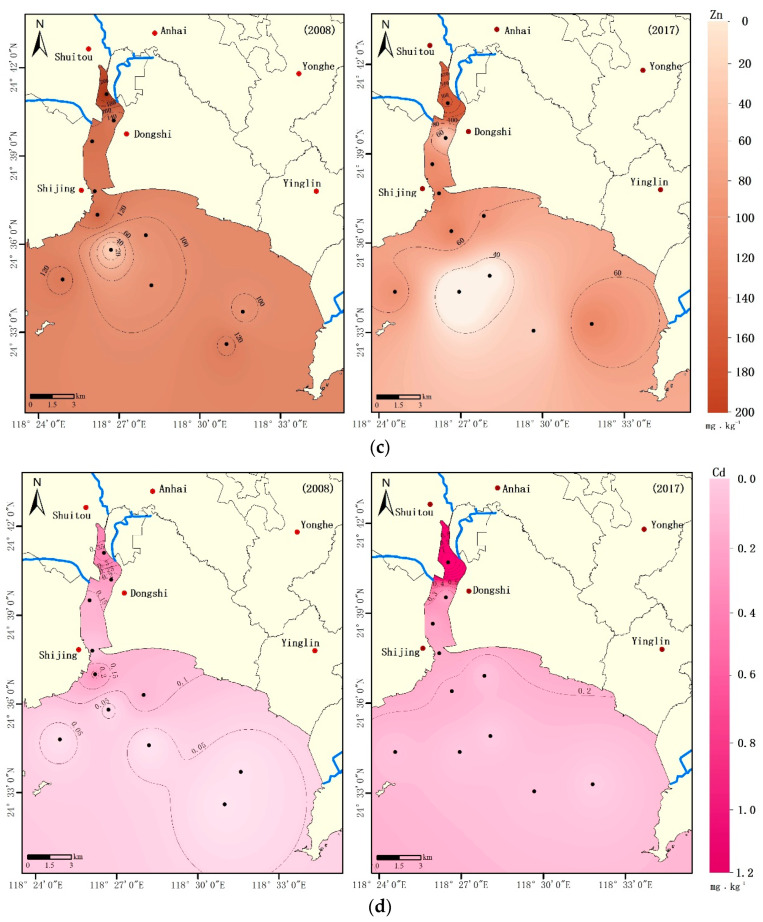

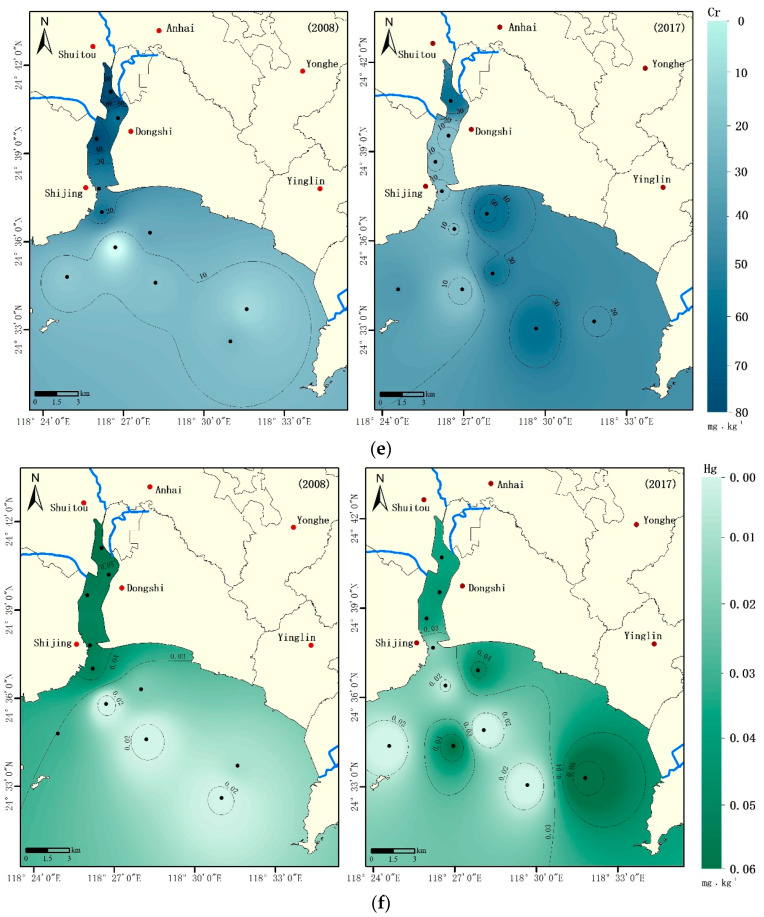

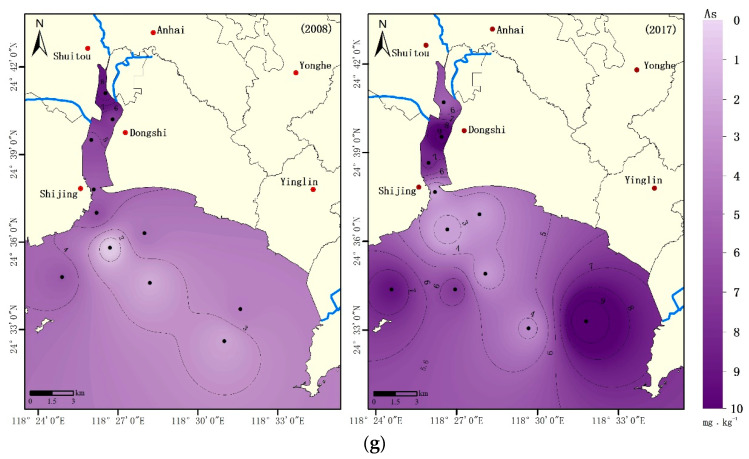
Spatial distributions of the heavy metals (**a**) Cu, (**b**) Pb, (**c**) Zn, (**d**) Cd, (**e**) Cr, (**f**) Hg, and (**g**) As concentrations in Weitou Bay surface sediment.

The spatial distributions of heavy metal concentrations are shown in Figure 3. Concentrations of heavy metals in Weitou Bay are related to several factors and are indeed influenced by anthropogenic activity and hydrodynamic conditions. In 2008, the areas with high concentration of all heavy metals in sediment were located in inner Weitou Bay, and the spatial distribution exhibited a decreasing trend from north to south in the bay. In general, higher heavy metal concentrations in the inner bay are caused by large amounts of terrestrial pollution input by the three rivers flowing into the bay and sewage discharges from the coastal industrial zone. In addition, the weak hydrodynamic conditions in the inner bay prevent heavy metals from being diluted rapidly, resulting in their deposition in the sediment. Heavy metals discharged into the inner bay are partially deposited in sediments before they enter the outer bay with tidal currents. This could be one of the reasons why the mean heavy metal concentrations in the outer bay sediment were lower than those in the inner bay.

In 2017, two features of the spatial distribution of heavy metal concentrations were observed, including increasing concentrations in the outer bay and a shift of high concentration from the inner bay to the outer bay. High heavy metal concentrations were observed in the southeastern inshore area of the outer bay, where a new industrial center (including chemical fiber, printing and dyeing, electroplating, and other industries) was built after 2008. In contrast, except for Cd and As, heavy metal concentrations decreased in the inner bay. This improvement can be largely attributed to a series of remediation measures in the inner bay between 2008 and 2017, including upstream river regulation, sewage interception, and the closure of small polluting enterprises. For Cu and Zn, although the average concentrations decreased in both the inner and outer bay (Figure 2), higher concentrations were observed in the outer bay (Figure 3). Pb, Cr, and Hg exhibited similar changes in spatial distribution from 2008 to 2017 (Figure 2), as the average concentrations of these heavy metals decreased in the inner bay but increased in the outer bay. For Cd and As, overall increases in their average concentrations occurred throughout the bay; however, the rate of increase in the outer bay was much higher than that of the inner bay (Figure 2). From 2008 to 2017, the average Cd concentration increased by 136% (from 0.198 mg·kg^−1^ to 0.467 mg·kg^−1^) in the inner bay but increased by 249% (from 0.043 mg·kg^−1^ to 0.150 mg·kg^−1^) in the outer bay. Meanwhile, As increased by 12% in the inner bay and by 80% in the outer bay. 

### 3.2. Heavy Metal Sources

Previous studies have indicated that heavy metals in surface sediment originate from various sources and could be primarily affected by three factors: (1) pollutant emissions from urban development and human activity; (2) adsorption and sequestration with organic matter and sulfide; and (3) natural weathering and erosion of rocks [23,24,25]. Therefore, correlation analysis and PCA were conducted to explore the relationships between the seven heavy metals, TOC, and sulfide in the surface sediment. 

Before Pearson correlation analysis, the data were tested for normality. Only the data of five parameters (TOC, Pb, Zn, Hg, and As) had a normal distribution (*p* > 0.05). A log−transformation was applied to the other parameters (Sulfide, Cu, Cd, and Cr) to satisfy the conditions for the statistical tests. Statistically, these independent variables have mutual relationships, which is suitable for PCA, with a Kaiser‒Meyer‒Olkin (KMO) value of 0.625 (>0.5) and a Bartlett’s spherical test value of 0. 

Table 2 presents the results of the correlation analysis, indicating that Cu, Zn, Cr, Cd, and Hg have significant positive correlations with TOC, and that Cu, Cr, Cd, and Hg have significant positive correlations with sulfide. Chemical−specific surface adsorption, cation exchange reactions, and chelating reactions may play significant roles in the spatial distribution pattern of heavy metals in Weitou Bay. These reactions form metal organic complexes or precipitate metal sulfides, thereby promoting the migration of heavy metals from the water to the sediment. In a comparison of the ability of TOC and sulfides to control heavy metal distributions, Zn was more selectively absorbed by TOC than by sulfide. Moreover, Cu, Zn, and Hg had better correlations with TOC and sulfide. In contrast, Cr and Cd were more related to concentration by sulfide. There were significant correlations among Cu, Cd, and Cr. In addition, Cu, Zn and Hg had significant correlations, and there were remarkable correlations between As, Cd, and Hg. The relatively high correlations among the heavy metals indicate that they likely shared the same pollution source, geochemical behaviors, and mobility pathways, or at least shared one main origin.

The results of the PCA (Table 3) indicate that the first three principal components accounted for 81.727% of the total variance, which may play an important role in explaining the potential pollution source and other factors affecting the distribution of heavy metals. The first principal component (PC1) accounted for 49.936% of the total variance, exhibiting high positive loads from TOC (0.826), sulfide (0.821), Cu (0.832), Zn (0.700), Cd (0.769), Cr (0.697), Hg (0.773), and As (0.499). PC1 may reflect an anthropogenic origin, including industrial waste, agricultural source pollutants, and urban domestic sewage, which are the major contributors of heavy metals to marine sediments [12]. The spatial distributions of heavy metals in Weitou Bay indicate that the high concentration areas are located near the river mouth (inner bay) and along the coast of a concentrated industrial area, which is consistent with the large number of riverside or coastal industries, including printing and dyeing factories, tanneries, and electroplating factories. Wastewater from printing and dyeing factories contains a variety of heavy metal pollutants, including Cu, Zn, Cd, Cr, and Hg. Moreover, Cr, Zn, and Cu are commonly used in electroplating, and Cr is the main heavy metal pollutant in tanning facilities. Fertilizers and pesticides used for agriculture are generally the main sources of As and Hg [26,27,28,29,30]. In addition, the contributions from TOC and sulfide in PC1 reached 0.826 and 0.821, respectively, which further supports a scenario where organic carbon and sulfide play important roles in metal ion complexing. The release of metal ions accompanied by organic matter degradation and the binding and fixation by sulfur ions are also important sources of heavy metals in sediments.

The second principal component (PC2) explained 17.483% of the variance, with high contributions from Pb (0.931) and Cr (0.583). In this case, atmospheric deposition and precipitation were also significant factors that contributed to the sources of Pb and Cr. Some studies have shown that urban transportation is the main source of Pb and Cr, since alloy materials used in car bodies and yellow paint on road surfaces contain high concentrations of Cr, and vehicle emissions contain considerable amounts of Pb [22,31]. Pb in the environment is mainly derived from fossil fuel combustion [7]. After it is released through industrial combustion and automobile exhaust, Pb enters the water through atmospheric deposition and rainfall. In addition, port shipping is another important source of Pb in Weitou Bay. According to data from the annual Quanzhou City Statistics Bulletin on National Economic and Social Development, there were 0.33 million vehicles in Quanzhou in 2008, with a total annual port capacity of 72.24 million tons and a total social electricity consumption of 26.9 billion kWh. By 2017, the number of vehicles reached 1.35 million, the annual port throughput was 129.86 million tons, and the total social power consumption was 47.0 billion kWh. These data indicate that the fossil fuel consumption in Quanzhou City increased from 2008 to 2017, which can lead to the doubling of Pb contents in the surface sediments of Weitou Bay, which is consistent with the data obtained in this study.

The third principal component (PC3) explained 14.308% of the variance, with the largest contribution from As, whose spatial variations were represented by relatively low RSDs among the studied metals. Moreover, the mean concentration of As was similar to the environmental background value. Based on the above analysis, PC3 indicates a natural source, such as rock weathering or erosion. In addition, the PC1 value was also highly correlated with As. From the results of the correlation analysis, As was significantly correlated with Hg, which indicates that As in the Weitou Bay surface sediment is affected by both human activity and natural processes.

### 3.3. Potential Ecological Risks of Heavy Metals

The results of the potential ecological risk assessment are summarized in Table 4. In 2008 and 2017, the mean $E_{r}^{i}$ values exhibited decreasing orders of Cd (54.0) > Hg (21.4) > Cu (8.6) > As (6.5) > Zn (1.4) > Pb (0.9), and Cd (126.4) > Hg (19.3) > As (8.8) > Cu (3.4) >Pb (1.3) > Zn (0.8), respectively. In both 2008 and 2017, Cd was the most harmful metal, followed by Hg. These two elements accounted for more than 80% of the comprehensive *RI*, indicating that they are the main potential ecological risk factors in Weitou Bay.

The individual element potential ecological risk indices indicate that, in Weitou Bay, the $E_{r}^{i}$ values of most of the heavy metals were less than 40, reflecting an overall low ecological risk status, except for Cd. The $E_{r}^{i}$ contribution rates to the *RI* by Cd in 2008 and 2017 were 57.6% and 78.5%, respectively, which were significantly higher than those of the other heavy metals. The maximum $E_{r}^{i}$ value for Cd increased from 182.9 (2008) to 561.9 (2017), indicating that the potential ecological risk for Cd increased from ‘very high’ to ‘disastrous’. 

**Figure 4:**
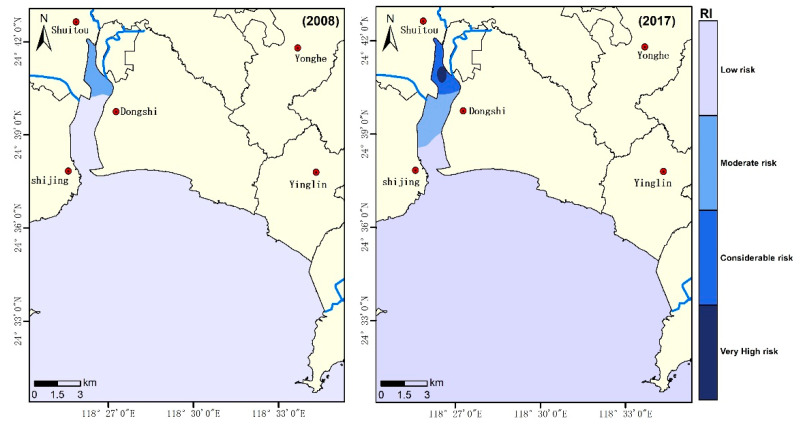
Spatial distribution of *RI* in Weitou Bay.

The spatial distribution of *RI* values generally decreased from north to south due to the intense industrial and other human activities located along the coast of the inner bay (Figure 4). Even with 10 years of economic development, the *RI* only increased significantly in the inner bay. In 2008, 18% of the sampling sites had moderate ecological risks (mostly in the inner bay), while the others had low ecological risks. The situation was worse in 2017, with 18% of the sampling sites having moderate ecological risks and 9% having very high ecological risks due to Cd pollution. As shown in Table 3, PC1 (49.936% of the total variance) had a high contribution from Cd (0.769), indicating that anthropogenic origins are the major source of Cd pollution. Since there are many factories that emit heavy metal pollutants, reducing industrial pollution should attract special attention for mitigating pollution and increasing ecological restoration in Weitou Bay.

## 4. Conclusions

Based on the concentrations of TOC, sulfides, and heavy metals in the surface sediment samples collected from Weitou Bay in 2008 and 2017, we analyzed the spatiotemporal variations in heavy metal contents in the surface sediment and further assessed the pollution sources and potential ecological risks. High concentrations of pollutants were generally located near the river mouths (inner bay) and along the coast of the industrial area and shifted from the inner bay to the outer bay from 2008 to 2017. This indicates that heavy metals are associated with anthropogenic activity and hydrodynamic conditions in the bay. The average heavy metal concentrations were lower than their background values, except those of Cu, Zn, and Cd in 2008 and that of Cd in 2017.

Multivariate analyses indicate that Cu, Zn, Cd, Cr, and Hg were mainly derived from industrial pollution, Pb predominantly originated from traffic emissions and industrial combustion, and agricultural activity and natural processes were the major sources of As. Moreover, physicochemical interactions with organic matter and sulfide may play significant roles in the distribution of heavy metals in Weitou Bay.

In general, Weitou Bay suffers from low to moderate heavy metal ecological risk, except for a small region that suffers from very high risk. From 2008 to 2017, the potential ecological risk of heavy metals in the surface sediments increased in the inner of Weitou Bay, which should be treated as the prior ecological protection areas. Cd was a large contributor (57.6% and 78.5% in 2008 and 2017, respectively) to the RI, and was the most serious of the polluting metals in the 2017 sediment samples, followed by Hg. 

Long−term heavy metal monitoring, particularly of Cd and Hg, should be conducted to obtain real−time tracking of potential ecological risks in this region. Controlling industrial effluent discharge is the most effective measure for improving the marine environment. Overall, this study provides important guidance for pollution control measures and ecological restoration in Weitou Bay.

## Figures and Tables

**Table 1:** Heavy metal concentrations in the surface sediment of Weitou Bay.

Year	Value	TOC (%)	Sulfide	Cu	Pb	Zn	Cd	Cr	Hg	As
			(mg·kg^−1^)							
2008	Min	0.35	12.4	3.5	1.8	23.4	0.016	2.61	0.017	1.30
	Max	1.84	841.0	155.0	11.9	213.0	0.384	78.40	0.060	8.50
	Mean−whole	0.94	179.6	38.7	6.6	118.6	0.113	20.87	0.034	4.10
	Mean−inner	1.33	361.3	73.7	9.6	149.0	0.198	38.22	0.048	5.54
	Mean−outer	0.62	28.2	9.5	4.2	93.3	0.043	6.41	0.022	2.93
	SD	0.46	289.0	44.9	3.8	45.2	0.113	23.08	0.015	1.90
	RSD	0.5	1.6	1.2	0.6	0.4	1.0	1.1	0.4	0.5
2017	Min	0.29	23.5	3.1	0.1	20.8	0.123	5.20	0.012	2.10
	Max	1.24	130.5	62.8	26.3	161.0	1.180	56.30	0.054	10.00
	Mean−whole	0.60	207.6	15.2	10.2	62.9	0.265	21.77	0.030	5.60
	Mean−inner	0.86	171.6	25.8	7.5	85.2	0.467	15.78	0.034	6.23
	Mean−outer	0.46	60.4	9.13	11.8	50.1	0.150	25.19	0.029	5.27
	SD	0.31	377.4	16.3	9.3	36.9	0.306	17.23	0.015	2.70
	RSD	0.5	1.8	1.1	0.9	0.6	1.2	0.8	0.5	0.5
Background		/	/	22.4	39	83.6	0.06	40.70	0.063	6.38

Min, max, mean−whole are the minimum, maximum, and average concentrations of the entire Weitou Bay, respectively. Mean−inner and mean−outer are the average concentrations of the inner and outer Weitou Bay, respectively. SD and RSD are the standard deviation and relative standard deviation of the entire Weitou Bay, respectively. Background values are the concentrations of heavy metals in the coastal soils of Fujian Province. “/” indicates no available comparison data.

**Table 2:** Pearson correlation matrix for heavy metals, TOC, and sulfide in the surface sediment of Weitou Bay.

	TOC	Sulfide	Cu	Pb	Zn	Cd	Cr	Hg	As
TOC	1								
Sulfide	0.671 **	1							
Cu	0.690 **	0.562 **	1						
Pb	−0.053	0.293	0.013	1					
Zn	0.866 **	0.407	0.645 **	−0.139	1				
Cd	0.465 *	0.773 **	0.583 **	0.088	0.235	1			
Cr	0.459 *	0.556 **	0.449 *	0.668 **	0.407	0.502 *	1		
Hg	0.530 *	0.497 *	0.656 **	0.070	0.481 *	0.487 *	0.399	1	
As	0.160	0.286	0.305	−0.117	0.099	0.554 **	0.250	0.615 **	1

** *p*< 0.01; * *p*< 0.05.

**Table 3:** Results of the principal component analysis for heavy metals, TOC, and sulfide in the surface sediment of Weitou Bay.

	PC1	PC2	PC3
TOC	0.826	−0.277	−0.396
Sulfide	0.821	0.214	0.000
Cu	0.832	−0.199	−0.095
Pb	0.183	0.931	−0.164
Zn	0.700	−0.395	−0.503
Cd	0.769	0.116	0.389
Cr	0.697	0.583	−0.150
Hg	0.773	−0.142	0.271
As	0.499	−0.120	0.771
Eigenvalue	4.494	1.573	1.288
% of variance	49.936	17.483	14.308
Cumulative %	49.936	67.419	81.727

**Table 4:** Potential ecological risks for heavy metals in the surface sediment of Weitou Bay.

Year	Station	$E_{r}^{i}$							*RI*
		Cu	Pb	Zn	Cd	Cr	Hg	As	
2008	Min	0.8	0.2	0.3	7.8	0.1	10.8	2.0	27.0
	Max	34.6	1.5	2.5	182.9	3.9	38.1	13.3	276.0
	Mean	8.6	0.9	1.4	54.0	1.0	21.4	6.5	93.8
	Low risk (%)	100	100	100	55	100	100	100	82
	Moderate risk (%)	0	0	0	18	0	0	0	18
	Considerable risk (%)	0	0	0	18	0	0	0	0
	Very high risk (%)	0	0	0	9	0	0	0	0
	Disastrous risk (%)	0	0	0	0	0	0	0	/
2017	Min	0.7	0.0	0.2	58.6	0.3	7.6	3.3	78.8
	Max	14.0	3.4	1.9	561.9	2.8	34.3	15.7	612.2
	Mean	3.4	1.3	0.8	126.4	1.1	19.3	8.8	161.0
	Low risk (%)	100.0	100.0	100.0	0.0	100.0	100.0	100.0	73
	Moderate risk (%)	0	0	0	55	0	0	0	18
	Considerable risk (%)	0	0	0	36	0	0	0	0
	Very high risk (%)	0	0	0	0	0	0	0	9
	Disastrous risk (%)	0	0	0	9	0	0	0	/

“/” indicates no available comparison classification.
